# Galectin-9 Facilitates Epstein-Barr Virus Latent Infection and Lymphomagenesis in Human B Cells

**DOI:** 10.1128/spectrum.04932-22

**Published:** 2023-01-09

**Authors:** Jing-xiao Xu, Rong Zhang, Dai-jia Huang, Ying Tang, Li-qin Ping, Bi-jun Huang, Hui-qiang Huang, Pierre Busson, Jiang Li

**Affiliations:** a State Key Laboratory of Oncology in South China, Collaborative Innovation Center for Cancer Medicine, Department of Biotherapy, Sun Yat-sen University Cancer Center, Sun Yat-sen University, Guangzhou, China; b State Key Laboratory of Oncology in South China, Collaborative Innovation Center for Cancer Medicine, Department of Endoscopy and Laser, Sun Yat-sen University Cancer Center, Sun Yat-sen University, Guangzhou, China; c Institute of Tumor, Guangzhou University of Chinese Medicine, Guangzhou, China; d State Key Laboratory of Oncology in South China, Collaborative Innovation Center for Cancer Medicine, Department of Medical Oncology, Sun Yat-sen University Cancer Center, Sun Yat-sen University, Guangzhou, China; e State Key Laboratory of Oncology in South China, Collaborative Innovation Center for Cancer Medicine, State Key Lab of Oncology in South China, Sun Yat-sen University Cancer Center, Sun Yat-sen University, Guangzhou, China; f CNRS-UMR 9018-METSY, Gustave Roussy and Université Paris-Saclay, Villejuif, France; Institute of Molecular Biology, Academia Sinica

**Keywords:** EBV, galectin-9, LCL, B-cell lymphoma, EBNA1, B cell immortalization, B-cell lymphomas, Epstein-Barr virus

## Abstract

The immune regulator galectin-9 (Gal-9) is commonly involved in the regulation of cell proliferation, but with various impacts depending on the cell type. Here, we revealed that Gal-9 expression was persistently increased in Epstein-Barr virus (EBV)-infected primary B cells from the stage of early infection to the stage of mature lymphoblastoid cell lines (LCLs). This sustained upregulation paralleled that of gene sets related to cell proliferation, such as oxidative phosphorylation, cell cycle activation, and DNA replication. Knocking down or blocking Gal-9 expression obstructed the establishment of latent infection and outgrowth of EBV-infected B cells, while exogenous Gal-9 protein promoted EBV acute and latent infection and outgrowth of EBV-infected B cells at the early infection stage. Mechanically, stimulator of interferon gene (STING) activation or signal transducer and activator of transcription 3 (STAT3) inhibition impeded the outgrowth of EBV-infected B cells and promotion of Gal-9-induced lymphoblastoid cell line (LCL) transformation. Accordingly, Gal-9 expression was upregulated by forced EBV nuclear antigen 1 (EBNA1) expression in 293T cells *in vitro*. Clinical data showed that Gal-9 expression in B-cell lymphomas (BCLs) correlated positively with EBNA1 and disease stage. Targeting Gal-9 slowed LCL tumor growth and metastasis in xenografted immunodeficient mice. These findings highlight an oncogenic role of Gal-9 in EBV-associated BCLs, indicating that Gal-9 boosts the transformation of EBV-infected B cells.

**IMPORTANCE** The cross talk between Epstein-Barr virus (EBV) and the host cell transcriptome assumes important roles in the oncogenesis of EBV-associated malignancies. Here, we first observed that endogenous Gal-9 expression was persistently increased along with an overturned V-type change in antivirus signaling during the immortalization of EBV-transformed B cells. Upregulation of Gal-9 promoted the outgrowth and latent infection of EBV-infected B cells, which was linked to B-cell-origin tumors by suppressing STING signaling and subsequently promoting STAT3 phosphorylation. EBV nuclear antigen EBNA1 induced Gal-9 expression and formed a positive feedback loop with Gal-9 in EBV-infected B cells. Tumor Gal-9 levels were positively correlated with disease stage and EBNA1 expression in patients with B-cell lymphomas (BCLs). Targeting Gal-9 slowed the growth and metastases of LCL tumors in immunodeficient mice. Altogether, our findings indicate that Gal-9 is involved in the lymphomagenesis of EBV-positive BCLs through cross talk with EBNA1 and STING signals.

## INTRODUCTION

Epstein-Barr virus (EBV) is an oncogenic human gammaherpesvirus that is involved in the development of several malignancies, such as nasopharyngeal carcinoma (NPC), and a fraction of gastric cancers and B-cell lymphomas (BCL) (1). However, the mechanisms through which these malignancies develop are not completely understood. EBV has evolved mechanisms to alter the fate of infected host cells. They include the expression of multiple latent viral genes such as EBV nuclear antigens 1 to 6 (EBNA1 to -6) and latent membrane proteins 1 and 2 (LMP1 and 2) and the upregulation of host cell genes preventing cell death and apoptosis and favoring resistance to innate and adaptive immune cells (2, 3). Latent infection of B cells in asymptomatic carriers is a risk factor of fatal lymphoproliferation and lymphomagenesis when immune surveillance fails (4).

Galectin-9 (Gal-9), a β-galactoside-binding protein, has been identified as a ligand of immune receptor proteins, including Tim-3, CD45, and CD44, on various immune cell subsets (5–7). In addition to immune regulation, the function of Gal-9 is linked to cell proliferation and differentiation. It has been reported that Gal-9 binds Tim-3 to induce T-cell apoptosis but promotes outgrowth of myeloid leukemia stem cells (8, 9). Conversely, Gal-9 inhibits B-cell activation by binding to CD45 (6). It has been reported that Gal-9 expression is upregulated in EBV-associated NPC and that Gal-9 interacts with EBV-encoded LMP1 within NPC cells and exosomes derived from NPC cells (10). Our previous work showed that Gal-9 is associated with a poor prognosis in patients with NPC and induces tumor immune tolerance by mediating the expansion of myeloid-derived suppressor cells in a manner dependent on stimulator of interferon gene (STING) signaling (11). Thus, we hypothesize that Gal-9 can have an oncogenic role in the pathogenesis of EBV-associated tumors.

*In vitro*, EBV infection of primary human B cells leads to their growth transformation and serves as a model of the oncogenic mechanisms relevant to EBV-associated BCLs (12). Here, using *in vitro* EBV transformation of B cells as an experimental model, we first revealed a dynamic transcriptome alteration in EBV-infected B cells from the initiation of EBV infection to the stage of mature lymphoblastoid cell lines (LCL). We also found a sustained increase in Gal-9 mRNA expression. By blocking or forcing Gal-9 expression in the early stage of EBV infection, we gained evidence that Gal-9 facilitated the establishment of latent infection and the outgrowth of immortalized clones. Activating STING signals or inhibiting phosphorylated signal transducer and activator of transcription 3 (p-STAT3) in the early infection stage impaired Gal-9 promotion of EBV-infected B-cell outgrowth. In addition, Gal-9 expression at both the mRNA and protein levels was upregulated by EBNA1. A positive association between Gal-9 and EBNA1 expression, on the one hand, and tumor stage, on the other hand, was recorded in BCL patients. Moreover, targeting of Gal-9 slowed down the growth of LCL tumors in immunodeficient NCG mice.

## RESULTS

### Evolving patterns of transcription profiles for Gal-9 and other host cell gene sets during EBV-driven transformation.

**FIG 1:**
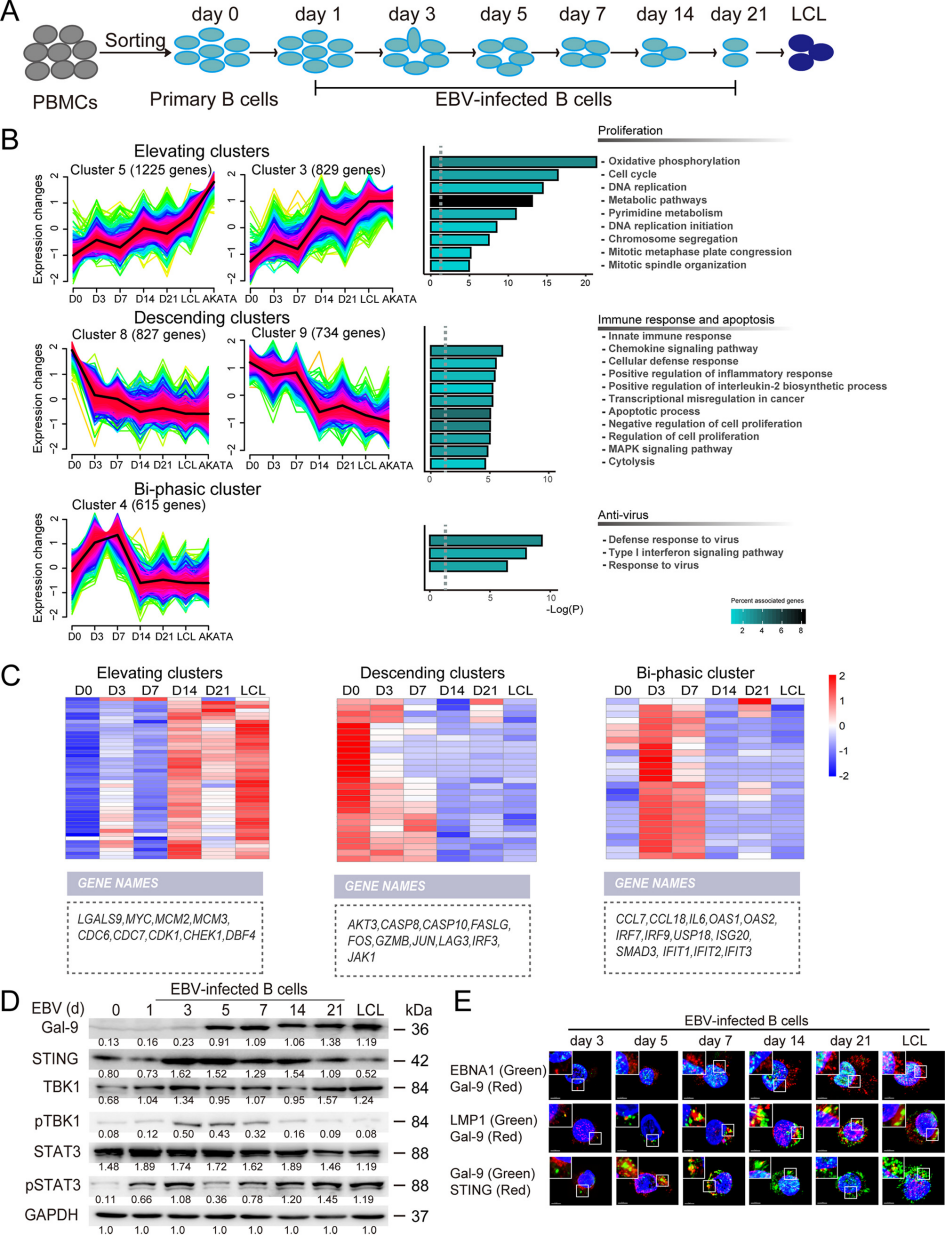
Evolution of the B-cell transcription profile following EBV infection and transformation. (A) Scheme of the timeline and stages of the EBV-mediated transformation of primary B lymphocytes. B cells were enriched from peripheral blood mononuclear cells (PBMCs) of healthy donors by negative selection using anti-CD3 beads. (B) Bioinformatics analysis of data from RNA deep sequencing performed at different stages (days 0, 3, 7, 14, and 21 and mature LCL stage) of EBV-driven transformation of primary B cells from one donor (and comparison with data from AKATA cells). The left panel displays the 3 temporal categories of RNA clusters: the continuously increasing elevating clusters, the continuously decreasing descending clusters, and the biphasic cluster. Clusters were generated using Mfuzz analysis (hypergeometric test; false-discovery rate [FDR], ≤0.05) The right panel shows the KEGG pathway enrichment of the corresponding clusters; adjusted *P* values are shown as bar plots after −log_10_ (P) transformation. (C) Heatmaps showing the distribution of genes related to the 3 temporal and functional categories highlighted in Fig. 1B (elevating, descending, and biphasic clusters) and the variations in their transcription profile during the transformation process. A few genes of each category are listed below the heatmaps. (D) Western blot showing the kinetics of the protein concentrations, including those of Gal-9, STING, TBK1, p-TBK1 STAT3, and p-STAT3, in EBV-transformed B cells at the indicated time points from day 0 to 21 days. Protein extracts from mature LCLs were included as controls (rightmost column). (E) Representative immunofluorescence images showing double staining of EBNA1 (green) and Gal-9 (red), LMP1 (green) and Gal-9 (red), and Gal-9 (green) and STING (red) in EBV-infected B cells at the indicated time points after EBV infection. Cell nuclei were stained with DAPI (blue). Scale bar = 4 μm.

To better understand the molecular mechanisms of B-cell transformation by EBV, we investigated the dynamics of transcriptome alterations occurring in primary B cells from the initial EBV infection stage to the mature LCL stage by RNA-sequencing (RNA-seq) (Fig. 1A and Fig. S1A to D in the supplemental material). By comparing the evolving transcriptome of infected B cells with the transcriptome of resting B cells, we observed a progressive increase in the size of the set of differentially expressed genes. This divergence reached an apex at the stage of mature LCLs. In contrast, mature LCL cells were closer to AKATA cells than resting B cells, as shown by the volcano plots of Fig. S1E. Nine gene clusters having specific patterns of transcriptional variations during B-cell transformation were identified by Mfuzz bioinformatics analysis (Fig. 1B and Fig. S1F and Table S3). Cluster 5 (1,225 genes) and Cluster 3 (829 genes) exhibited a pattern of evolution characterized by a sustained increase in their expression level throughout the transformation process. These gene cluster data were consistent with others’ reports using K-means analysis (3, 13). Therefore, they were designated “elevating clusters”. As shown by KEGG pathway analysis, the genes listed in these clusters mostly encoded proteins involved in pathways related to cell proliferation, including cell cycle regulation, DNA replication, and metabolism; in contrast, genes encoding proteins involved in pathways related to the immune response, chemokine signaling, and apoptotic processes were sorted into cluster 8 (827 genes) and cluster 9 (734 genes), which was also similar to the previous studies (3, 13). These two clusters have also been identified by others. They showed a consistent decrease in expression through the transformation process and therefore were designated “descending clusters”. Genes encoding proteins involved in the antiviral response and type I interferon signaling pathway were enriched in cluster 4 (615 genes), which was characterized as a “biphasic cluster” (Fig. 1B); this cluster was not identified in previous studies. Most genes that were sorted into elevating clusters were related to cell proliferation, such as *MYC*, *MCM2*, and *MCM3. LGALS9* had the same profile of evolution. Most genes from descending clusters, such as *AKT3*, *CASP10*, *FASLG*, and *FOS*, were related to the immune response and control of programmed cell death. Biphasic clusters contained genes related to antiviral signaling pathways, including *CCL7* and *18*, *IL6*, *OAS1* and *2*, and *SMAD3*. These genes were transiently upregulated at the early stage of infection (from days 0 to 7) but were consistently downregulated from day 7 until the stage of mature LCL (Fig. 1C). The kinetics of mRNA expression for *LGALS9* and genes downstream of STING signaling were confirmed by real-time reverse transcriptase PCR (RT-PCR) analysis (Fig. S2). We further revealed that the expression tendency of Gal-9, phosphorylated STAT3 (p-STAT3 - a key molecule in B-cell transformation and LCL proliferation) (14, 15), and STING signaling proteins such as the activated TBK1 were consistent with the proliferation (cluster 5 and 3) and antiviral (cluster 4) pathway models of primary B cells post-EBV infection (Fig. 1D). Interestingly, immunofluorescence staining showed Gal-9 colocalization with LMP1 and STING in the cytoplasm of EBV-infected B cells, but not with EBNA1 (in the nucleus) (Fig. 1E).

### Gal-9 facilitates early transformation of B cells post-EBV infection.

**FIG 2:**
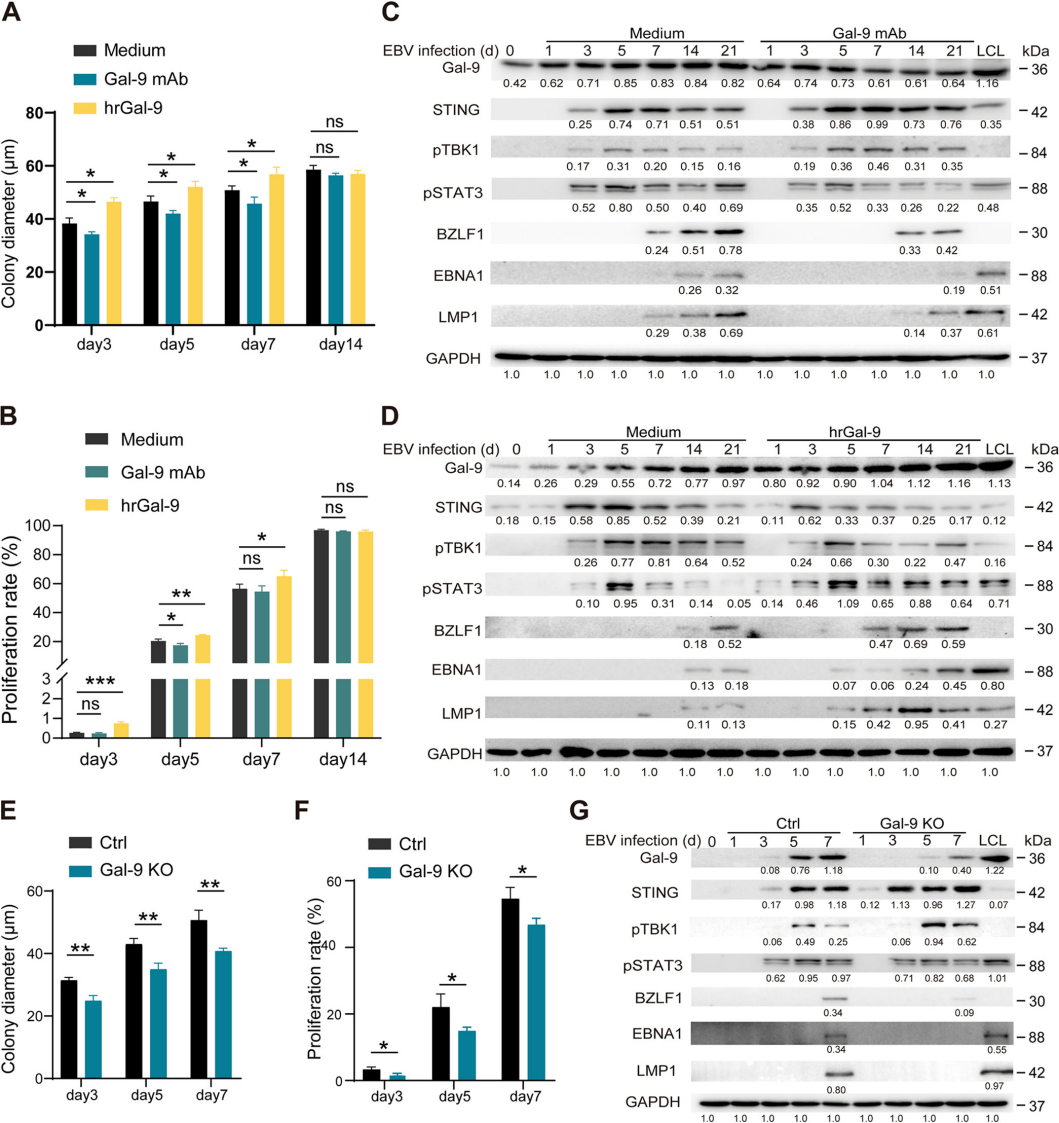
Gal-9 facilitates cell proliferation, colony formation, and latent viral protein expression at the early stage of EBV infection. (A and B) Statistical analysis of the mean diameter of EBV-infected B-cell colonies (A) and the proliferation of CSFE-labeled CD19^+^ cells (B) under the indicated treatment: medium, Gal-9 MAb, or hrGal-9. (C and D) Expression of Gal-9, STING, p-TBK1, p-STAT3, and the EBV proteins BZLF1, EBNA1, and LMP1 at different time points in EBV-infected B cells in the presence or absence of hrGal-9 or Gal-9 MAb. Nucleofection was used to knock down Gal-9 (Gal-9-KO) in primary B cells; Gal-9-KO and control (Ctrl) primary B cells were transformed by EBV 1 day after nucleofection. (E and F) Statistical analysis of the mean diameter of EBV-infected B-cell colonies (E) and the proliferation of CSFE-labeled CD19^+^ cells (F) at the indicated time points after EBV infection in Gal-9-KO and Ctrl EBV-infected B cells. (G) Expression of Gal-9, STING, p-TBK1, p-STAT3, and EBV proteins at different time points in Gal-9-KO and Ctrl EBV-infected B cells. Statistical significance in panels A, B, E, and F was determined using an unpaired Student’s *t* test; *, *P* < 0.05; **, *P* < 0.01; *P* < 0.05 was considered significant.

To investigate the function of Gal-9 in B cells during EBV infection, we first applied exogenous human recombinant Gal-9 (hrGal-9) and Gal-9 neutralizing antibody (Gal-9 MAb) to the culture medium, and we found that hrGal-9 accelerated B-cell proliferation of EBV-infected B cells at an early stage of the transformation process (days 3, 5, and 7) following EBV infection. At day 14, however, the colony formation process was identical in the various experimental groups. Accordingly, Gal-9 MAb attenuated EBV-transformed B-cell colony formation and proliferation (Fig. 2A and B and Fig. S3A and B). Moreover, hrGal-9 reduced the level of STING but increased the levels of p-STAT3 protein and the EBV proteins BZLF1, LMP1, and EBNA1, while Gal-9 MAb had a contrasting effect on those proteins (Fig. 2C and D). To further validate the physiological correction between exogenous Gal-9 and endogenous Gal-9, we knocked out endogenous Gal-9 in primary B cells by nucleofection, and we found that Gal-9 knockout in B cells obstructed the colony formation and proliferation of EBV-infected B cells at the early infection stage and the outgrowth of EBV-transformed cell clones (Fig. 2E and F and Fig. S3C and D). Moreover, knockout of Gal-9 induced higher levels of STING and pTBK1 but reduced levels of p-STAT3 protein and the EBV lytic protein BZLF1; the EBV latent proteins EBNA1 and LMP1 were still not detected at this stage (Fig. 2G). These data indicated that forced Gal-9 expression accelerated the early stages of EBV infection, onset of the latent phase, and B-cell transformation. Reciprocally, depletion of Gal-9 hampered the onset of viral latent infection and transformation in EBV-infected B cells.

### The STING signaling pathway is involved in the promotion of EBV-driven transformation of B cells by Gal-9.

**FIG 3:**
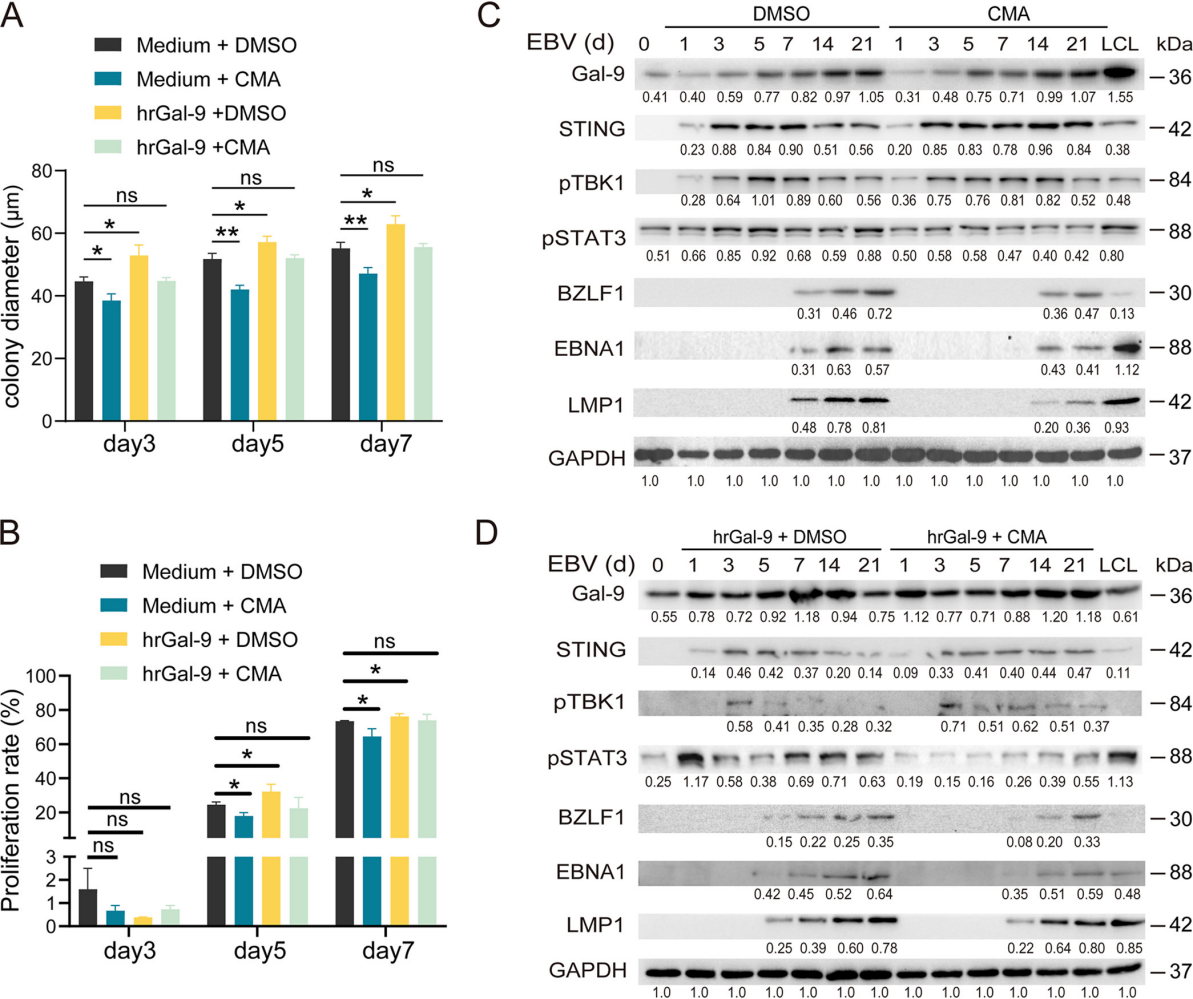
Activation of STING signaling suppresses the proliferation of EBV-infected B cells. (A and B) Statistical analysis of the mean diameter of EBV-infected B-cell colonies (A) and the proliferation of CSFE-labeled CD19^+^ cells (B) at the indicated time points after EBV infection in response to the indicated treatments: DMSO alone, CMA alone, DMSO plus hrGal-9, or CMA plus hrGal-9. (C and D) Protein levels of Gal-9, STING, p-TBK1, p-STAT3 and EBV proteins in B cells at the indicated time points after EBV infection in response to the indicated treatments. GAPDH was included as a control. Statistical significance in panels A and B was determined by an unpaired Student’s *t* test; *, *P* < 0.05; **, *P* < 0.01; *P* < 0.05 was considered significant.

Our previous study has shown that Gal-9 can physically interact with the STING protein and enhance its degradation (11). In the present study, we also observed that Gal-9 binding to STING occurred during the process of EBV-infected B-cell transformation (Fig. 1E). Moreover, during this process the expression levels of STING, p-TBK1, and p-STAT3 changed in opposite directions by Gal-9 knockout or addition of exogenous Gal-9 protein, as shown in Fig. 2C, F, and G. We wanted to further substantiate the idea that the contribution of Gal-9 to EBV-driven B cell transformation is linked to STING signaling suppression. In favor of our hypothesis, we found that colony formation of EBV-infected B cells was attenuated at an early stage (before day 7 following EBV infection) under treatment with the STING agonist 10-carboxymethyl-9-acrianone (CMA). Moreover, CMA weakened the capacity of hrGal-9 protein to promote EBV-infected B cell colony formation at an early stage of infection (Fig. 3A and B and Fig. S4). Interestingly, we found that CMA treatment restored the p-TBK1 level and decreased the p-STAT3 level as well as the levels of the EBV lytic and latent antigens BZLF1, LMP1, and EBNA1 after EBV infection (Fig. 3C and D). Overall, our data suggest that persistent activation of STING signaling impedes colony formation of EBV-infected B cells at the early stages of EBV infection and onset of the latent phase and simultaneously attenuates Gal-9 promotion of B-cell transformation upon EBV infection.

### Gal-9 promotes EBV-driven transformation of B cells partly through the STING-STAT3 signaling pathway.

**FIG 4:**
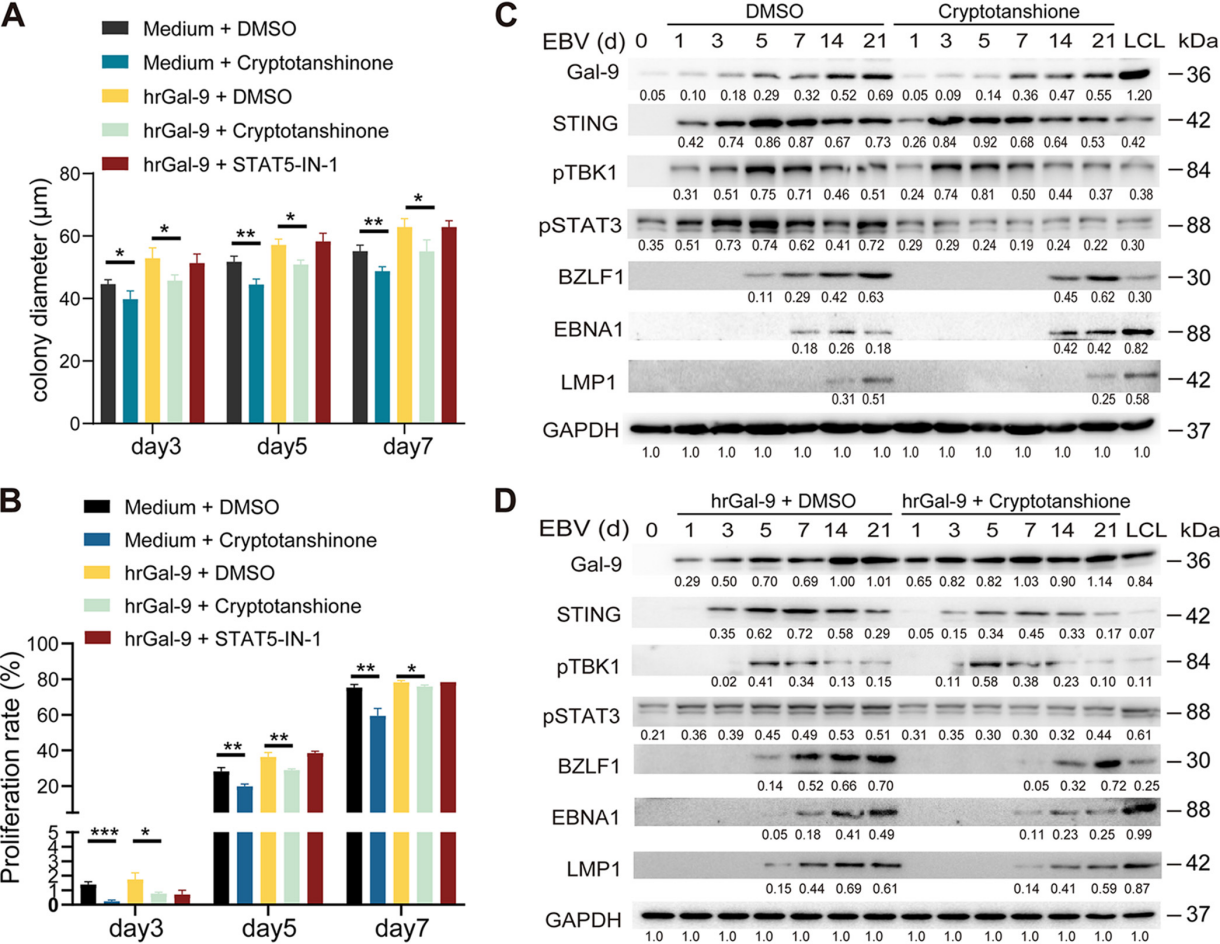
Inhibition of STAT3 signaling reduces the proliferation of infected B cells independently of Gal-9 treatment. EBV-infected B cells were treated with cryptotanshinone (a STAT3 inhibitor) alone, hrGal-9 alone, cryptotanshinone plus hrGal-9 or hrGal-9 plus STAT5-IN-1 (a STAT5 inhibitor). Vehicle (DMSO) alone served as a control. (A) Quantification of the mean diameter (five 100× fields) of EBV-infected B-cell colonies under each of the above-listed experimental conditions. (B) Statistical analysis of the proliferation of CSFE-labeled CD19^+^ cells at the indicated time points after EBV infection in response to the indicated treatments. (C and D) Western blot evaluation of the protein abundance of cellular Gal-9, STING, p-TBK1, p-STAT3, and viral BZLF1, EBNA1 and LMP1 in EBV-infected B cells at the indicated time points in response to either DMSO or p-STAT3 inhibitor alone (C) or combined with hrGal-9 (D). GAPDH expression was used as the loading control. The statistical significance in panels A and B was determined by an unpaired Student’s *t* test; *, *P* < 0.05; **, *P* < 0.01; *P* < 0.05 was considered significant.

We have previously reported that STING signaling could inhibit the phosphorylation of STAT3 via the STING-suppressor of cytokine signaling 1 (SOCS1)-STAT3 axis (16). In this study, we observed that cryptotanshinone, an inhibitor of STAT3, but not STAT5-IN-1, an inhibitor of STAT5, impeded the proliferation of EBV-infected B-cell colonies at an early stage of infection both in the absence and presence of hrGal-9 protein. Expression of the EBV latent proteins LMP1 and EBNA1 was also affected, but not of the lytic protein BZLF1 (Fig. 4A to D and Fig. S5). These data suggested that Gal-9 regulated the transformation of EBV-infected B cells partly through the Gal-9-STING-TBK1-STAT3 axis.

### EBNA1 upregulates Gal-9 expression.

**FIG 5:**
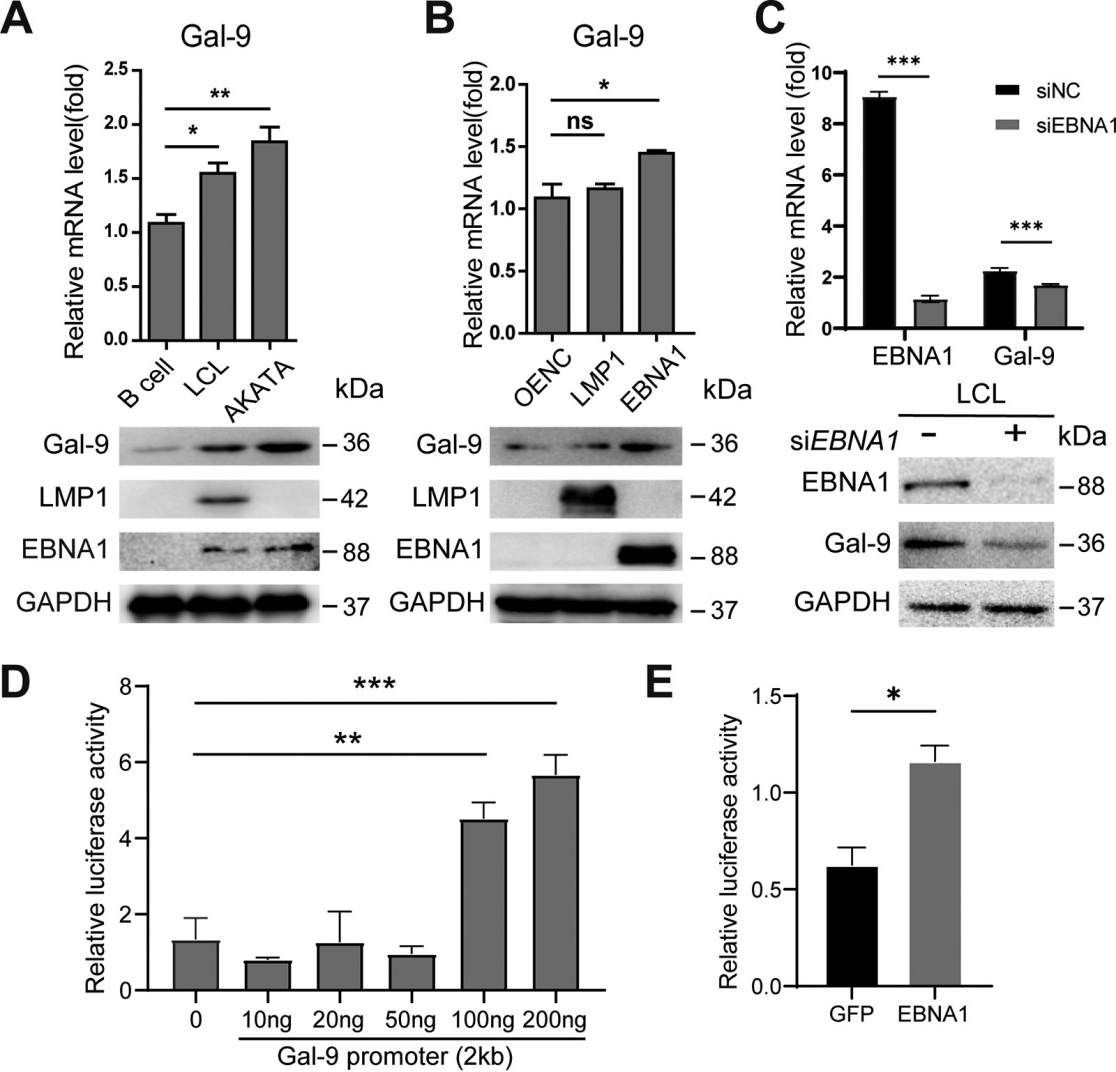
EBNA1 enhances Gal-9 expression at both the mRNA and protein levels. (A) Gal-9 mRNA and protein expression in EBV-negative primary B cells, fully transformed EBV-positive LCL cells, and EBV-positive AKATA cells. Assessment of mRNA and protein abundance was performed using real-time qPCR and Western blot analysis, respectively. (B) Gal-9 mRNA and protein expression in 293T cells transfected with genes encoding EBNA1 or LMP1 or with corresponding control vectors (OENC). GAPDH was included as a loading control. (C) LCLs were transfected with siRNA targeting EBNA1 or scrambled gene, and cells were harvested 48 h later and tested for the protein levels of EBNA1 and Gal-9 by immunoblotting. (D and E) C666 cells were transfected with a Gal-9 promoter reporter plasmid (a plasmid containing the Gal-9 promoter region fused to the luciferase gene) in different doses (D). C666 cells were also double-transfected with the Gal-9 promoter reporter plasmid plus the plasmid encoding EBNA1 or a control plasmid containing the *GFP* cDNA driven by the pLNCX2 vector (E) to detect transactivation of the Gal-9 gene. Comparisons between the two groups were conducted with an unpaired Student’s *t* test. *, *P* < 0.05; **, *P* < 0.01; *P* < 0.05 was considered significant.

Gal-9 mRNA transcription was maintained at a high level through the transformation process of EBV-infected B cells and retained in mature LCL cells (Fig. 1C). We hypothesized that Gal-9 expression might be induced in B cells by EBV infection. Here, we found that Gal-9 was highly expressed in EBV-positive LCLs and AKATA cells compared with EBV-negative primary B cells from healthy donors (Fig. 5A). We further demonstrated that Gal-9 expression was increased in 293T cells subjected to EBNA1 forced expression but not in 293T cells transfected with a control plasmid or subjected to LMP1 forced expression (Fig. 5B). Importantly, Gal-9 expression was reduced in LCL under administration of small interfering RNA (siRNA) targeting EBNA1 (Fig. 5C). Moreover, a luciferase reporter assay was utilized to confirm that EBNA1 induced Gal-9 transcription (Fig. 5D and E). Overall, these data indicated that EBNA1 could upregulate Gal-9 and that Gal-9 and EBNA1 formed a positive feedback loop to maintain the high level of Gal-9 expression in the process of EBV-infected B-cell transformation.

### Tumor Gal-9 abundance correlates with EBNA1 expression and tumor progression in BCL patients.

**FIG 6:**
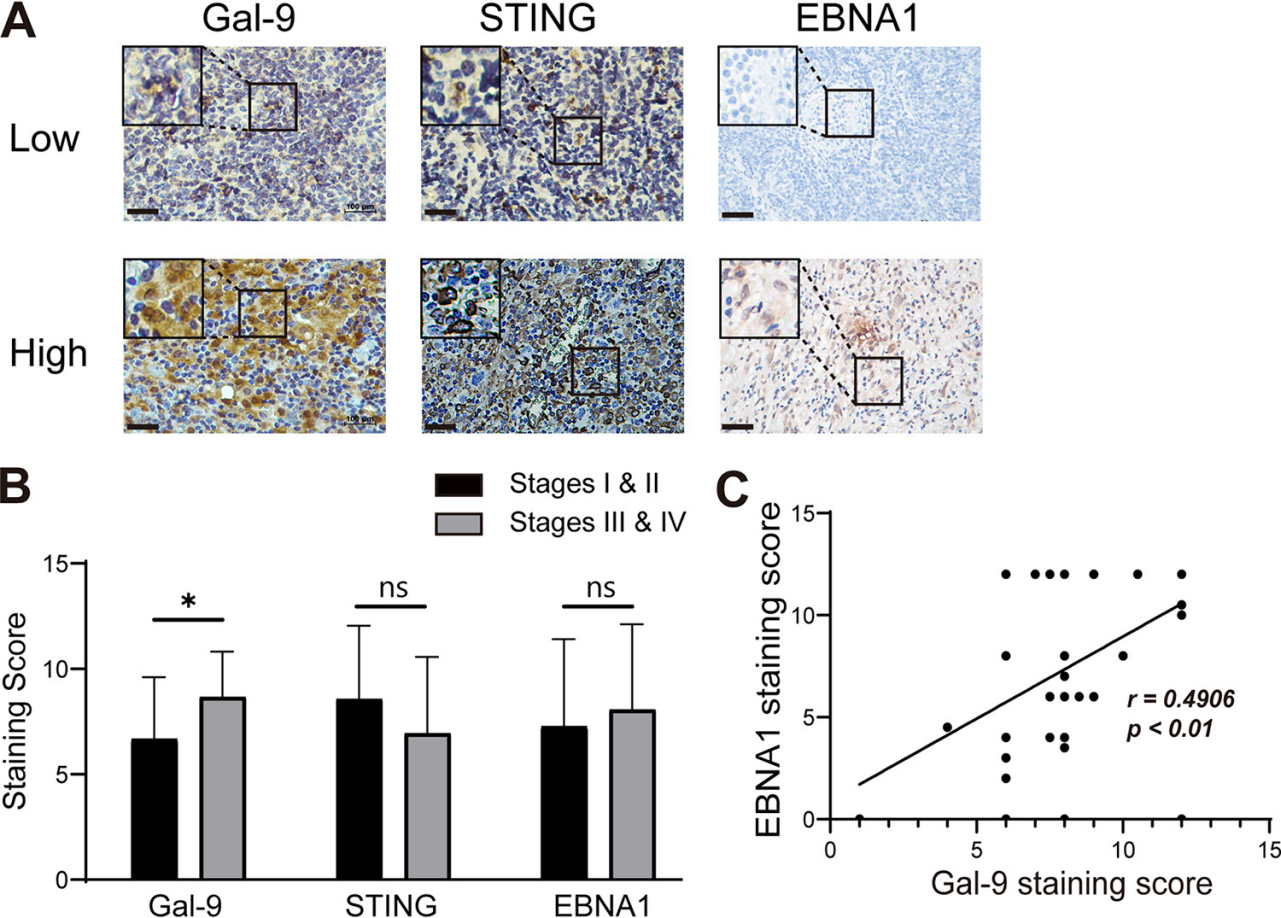
Intense Gal-9 expression in B-cell lymphoma is related to tumor stage and the abundance of EBNA1. The expression of Gal-9, STING, and the EBV protein EBNA1 was detected and semiquantitatively assessed by immunohistochemical staining in tumor sections from 66 BCL patients (43 of 66 patients had Ann Arbor stage information). (A and B) Representative images (A) and statistical analyses of staining scores (B) are shown for tumors distinguished by Ann Arbor stage. Scale bar = 100 μm; *n* = 43. (C) Spearman correlation analysis showing the correlation between the Gal-9 and EBNA1 scores (*n* = 61, *r* = 0.4906, *P* < 0.001).

To further determine the function of Gal-9 in the tumorigenesis of BCLs, we collected 66 tumor samples from BCL patients, including 31 patients with EBV-positive lesions, and the detailed patient information is shown in Table S1. We found that increased Gal-9 expression was linked to advanced disease stage (*n* = 43, Fig. 6A and B). Moreover, we found that the tumor Gal-9 level was positively correlated with EBNA1 expression in 66 patients with BCL (*r* = 0.4906, *P* < 0.01; Fig. 6C). Overall, these observations suggested that the upregulated Gal-9 expression contributed to tumor development in patients with EBV-positive BCL.

### Targeting Gal-9 reduces the growth of LCL tumors in NCG mice.

**FIG 7:**
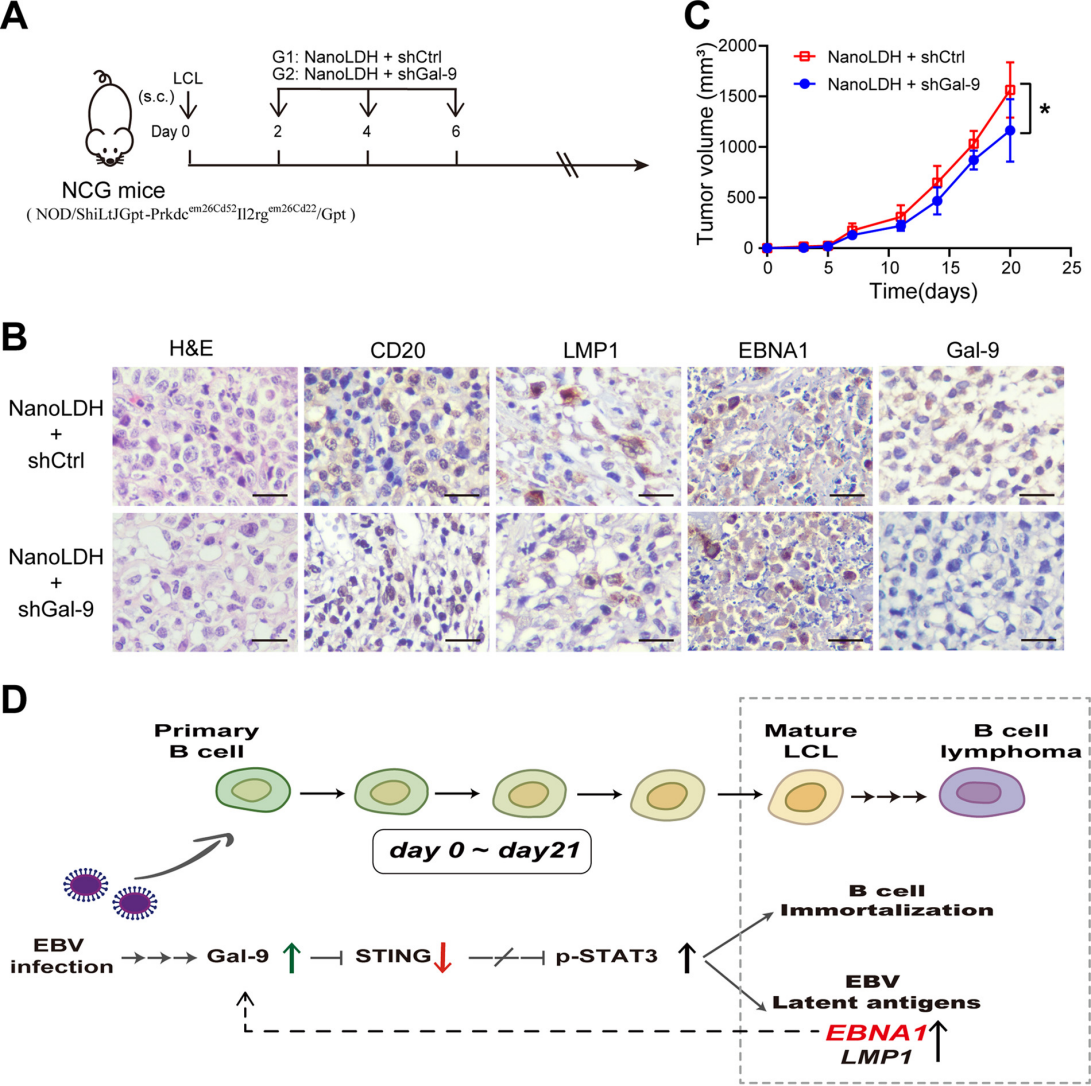
Targeting of Gal-9 reduces the growth of xenografted LCL tumors in NCG mice. (A) The treatment modalities of xenografted LCL tumors in NCG mice. Immunodeficient NCG mice were subcutaneously (s.c.) inoculated with 5 × 10^6^ mature LCL cells and exposed to shRNA plasmids loaded on LDH nanoparticles (NanoLDH) on days 2, 4, and 6 after tumor cell inoculation. The treatments were implemented as follows: G1 (*n* = 6), NanoLDH plus shCtrl, intratumor injection; G2 (*n* = 6), NanoLDH plus shGal-9 plasmid, intratumor injection. (B) The expression of CD20, EBNA1, LMP1, and Gal-9 was assessed in tumor tissue sections by IHC. Representative IHC images obtained with the indicated antibodies are displayed in the upper and lower panels for LCL tumors treated with shGal-9 or shCtrl plasmids, respectively. Scale bar = 100 μm. (C) Tumor growth kinetics were assessed by serial measurements of tumor volumes at successive time points. (D) Cartoon sketch summarizing the role of Gal-9 in the transformation and lymphomagenesis of EBV-infected B cells. The statistical significance in panel B was determined by an unpaired Student’s *t* test; *, *P* < 0.05; **, *P* < 0.01; *P* < 0.05 was considered significant.

LCL tumor xenografts in immunodeficient mice are a commonly used *in vivo* model of EBV-positive B-cell lymphomas (17, 18). To investigate whether Gal-9 is a potential therapeutic target in EBV-related BCLs, we examined xenografted LCL tumors in NCG (NOD/ShiLtJGpt-Prkdc^em26Cd52^Il2rg^em26Cd22^/Gpt) mice. Mice bearing tumors resulting from subcutaneous injections of mature LCL cells were treated with NanoLDH-loaded plasmids encoding short hairpin RNA (shRNA) targeting Gal-9 (NanoLDH-shGal-9) or control plasmids (NanoLDH-shCtrl), as shown in Fig. 7A. Pathological analysis showed that the LCL tumors displayed a phenotype characteristic of EBV+CD20^+^ B-cell lymphomas, with LMP1 and EBNA1 expression as well as Gal-9 expression, which were retained in the presence of shCtrl. In contrast, LCL Gal-9 expression could be blocked by shGal-9 *in vivo* and *in vitro* (Fig. 7B and Fig. S6A). We found that primary and metastatic (in the ipsilateral axilla and groin) LCL tumor growth was significantly slower in NanoLDH-shGal-9-treated mice than in NanoLDH-shCtrl-treated mice (Fig. 7C). Accordingly, the metastatic tumor numbers and weights of tumors in the NanoLDH-shGal-9-treated mouse group were lower than those in the control group (Fig. S6B). These data indicated that Gal-9 might provide a potential therapeutic target in EBV+BCLs.

## DISCUSSION

In the present study, we investigated the dynamics of transcriptome changes induced in primary B cells by EBV infection from the immediate postinfection stage to the stage of mature LCLs. *LGALS9* was one gene that underwent long-term upregulation following EBV infection. Using EBV transformation of primary B cells as an *in vitro* model, we showed that knockdown of endogenous Gal-9 or addition of exogenous Gal-9 impacted the establishment of EBV latent infection and the B-cell transformation process. The contribution of Gal-9 to this process involved a cross talk with STING signaling and the EBV antigen EBNA1. Our clinical data and investigations using a murine model provided further evidence of the involvement of Gal-9 in the lymphomagenesis of EBV-related BCLs.

Galectins are a family of evolutionarily conserved glycan-binding proteins involved in multiple cellular processes, such as apoptosis, proliferation, migration, and angiogenesis (19). Among them, Gal-9, Gal-1, and Gal-3 have been shown to control cell proliferation in various cell types, including epithelial and hematopoietic cells (20, 21). Moreover, Gal-1, -3, and -9 play a role in the emergence and progression of different types of leukemia, including acute myeloid leukemia, acute promyelocytic leukemia, B-cell lymphoblastic leukemia, adult T-cell leukemia, and chronic lymphocytic leukemia (22–24). In myeloid and T-cell-type leukemias, Gal-9 promotes leukemic cell proliferation by binding to its receptor Tim-3, which is usually overexpressed on the surface of leukemic stem cells. This leads to the activation of signaling cascades such as the phosphatidylinositol 3 (PI3) kinase/mammalian target of rapamycin (mTOR) and hypoxia-inducible factor 1 (HIF-1) pathways (25). In addition to its cell-autonomous functions in oncogenesis, Gal-9 can also promote human malignancies through its various immunosuppressive activities. Extracellular Gal-9 can induce T cell apoptosis, impair B-cell activation, and stimulate the expansion of myeloid-derived suppressor cells by binding to various membrane receptors, such as CD45, CD44, and TLR-4, in addition to its primary receptor Tim-3 (6, 7, 26, 27). Here, we first found that endogenous Gal-9 expression was persistently increased during the process of LCL transformation. We further showed that knockdown of endogenous Gal-9 or addition of a Gal-9 MAb in the culture medium at an early infection stage impaired the proliferation and colony formation of EBV-infected B cells (before 14 days) and impeded the acute and latent infection. Reciprocally, the addition of exogenous hrGal-9 protein promoted outgrowth of EBV-infected B cells and establishment of the acute/latent infection phases. These data indicated that Gal-9 likely assumed an important role in lymphomagenesis of EBV-positive BCLs. In several human malignancies, high expression of Gal-9 indicates a poor prognosis, which is linked to a large extent to its role in tumor immune escape (27–29). Our study showed that high expression of Gal-9 can also play a critical role in the regulation of cell proliferation and differentiation.

EBV infection is known to contribute to the oncogenesis of EBV-positive BCLs such as endemic Burkitt’s lymphomas, a fraction of Hodgkin lymphomas and diffuse large B-cell lymphomas (DLBCLs) (30–32). Use of the LCL transformation model and repeated RNA-seq analyses following the transformation process unveiled distinct kinetics for genes encoding latent viral proteins and host cell genes encoding proteins related to the antiviral response, such as STING and its downstream targets, including members of the interferon-stimulated gene (ISG) and suppressor of cytokine signaling (SOCS) families. These genes showed a trend of increased expression during the initial infection stage (days 0 to 7) and decreased expression from day 7 to the stage of mature LCLs (Fig. S2). The establishment of latent infection is a key step for EBV-infected cells to escape the host immune system and sometimes contribute to the onset of an EBV-positive tumor (33, 34). Our sequential RNA-seq analyses of EBV-infected B cells have shown that most genes undergoing long-term activation upon EBV infection are related to cell proliferation and survival, including cell cycle progression, DNA replication and repair, mitotic nuclear division, and the immune suppression pathway. This is consistent with the known effects of latent EBV products, for example, LMP1, which activates canonical and noncanonical NF-κB pathways, and EBNAs, which induce many cell cycle and antiapoptotic genes (for example, BCL-2) (2, 35). In addition to these transcription changes, we observed Gal-9 colocalization with STING in EBV-infected B cells. Moreover, the increase in Gal-9 expression occurred concomitantly with the decline in STING levels during the 3-week process, leading to fully transformed LCLs following EBV infection of primary B cells. We have previously reported that Gal-9 enhances the TRIM-29-mediated K48 ubiquitination of STING in epithelial NPC and myeloid cells through a direct protein-protein interaction (11). Here, we report that enhancing STING signaling during B-cell transformation restrains the latent infection and outgrowth of LCLs even in the presence of exogenous Gal-9. STING is a central signaling pathway in the regulation of the innate immune response to DNA virus and pathogenic microorganism infections. Activation of STING provokes type I interferon (IFN) signaling in innate immune cells and subsequently activates T cell immunity (36, 37). Recent studies have shown that inactivation of STING is linked to immune tolerance in cancers (38). In addition, foreign or endogenous small DNA molecules usually trigger autophagy resulting from activation of cGAS-STING pathway-mediated autophagy. Recent studies have shown that STING activation favors T cell apoptosis (39, 40). Consistently, our findings support the idea that the outgrowth and establishment of latent infection in EBV-infected B cells require the inactivation of STING signals. In our previous study, we revealed that deactivation of STING signaling in tumor cells increases p-STAT3 signaling, resulting in interleukin-6 (IL-6) and IL-1β production in the tumor microenvironment (16). STAT3 is constitutively active in EBV-related B and epithelial malignant cells and drives cell proliferation, invasion, and angiogenesis (15, 41). Here, we found that EBV-induced B-cell proliferation and colony formation were impeded when EBV-infected B cells were treated with a STAT3 inhibitor. Exogenous Gal-9 increased p-STAT3 levels, whereas activation of STING signaling with an agonist decreased p-STAT3 levels during LCL transformation.

We observed that Gal-9 was upregulated in EBV+LCL and B-cell lymphoma cell lines, and we further confirmed that forced EBNA1 expression upregulated Gal-9 expression in 293T cells, whereas knockdown of EBNA1 expression downregulated Gal-9 expression in LCL cells. Numerous studies have suggested that EBNA1 controls the transcription of viral latency genes and enhances the survival of infected cells and the transformation of primary B lymphocytes (42, 43). Here, we demonstrated that EBNA1 induced Gal-9 transcription in C666 cells. In addition, we found by *in silico* investigations in the Footprint DB database that two regions of the *Gal-9* gene promoter were potential EBNA1 binding sites—region 1 (P1: −274 bp to ~−258 bp) and region 2 (P2: −1692 bp to ~–1674 bp)—(Fig. S7A). However, the chromatin immunoprecipitation-quantitative PCR (ChIP-qPCR) analysis data did not strongly support EBNA1 binding to the P1 or P2 loci of the Gal-9 promoter compared with the known EBNA1 binding site on chromosome 11.1 (44) (Fig. S7B). To further demonstrate the role of Gal-9 in the lymphomagenesis of BCLs, we examined 66 samples from patients with BCL. In this series of BCL clinical specimens, we observed a correlation of tumor Gal-9 abundance with tumor stage and level of EBNA1 expression, but no influence of Gal-9 abundance on disease outcome (data not shown) in this small patient series. Our data support the concept of a positive feedback loop between Gal-9 and EBNA1. Therefore, inhibition of Gal-9 signaling may be beneficial to control EBV+ tumor growth. In this study, we found that targeting Gal-9 could delay LCL tumor growth and metastasis in NCG mice. This result supports the idea that Gal-9 might be a potential therapeutic target in EBV+BCLs. This might be a realistic perspective since blocking Gal-9/Tim-3 signaling has been reported as a possible approach for immunotherapy of some solid tumors (45). Overall, our findings reveal the first evidence of a novel role of Gal-9 in the pathogenesis of EBV-associated BCLs, involving the Gal-9/STING/STAT3 molecular axis and a positive feedback loop between the host cell gene Gal-9 and the EBV latent protein EBNA1 (Fig. 7D).

## MATERIALS AND METHODS

### Patients and cell lines.

Paraffin-embedded tumor tissues were collected from 66 patients with B-cell lymphoma—9 patients with Hodgkin lymphoma, 54 patients with non-Hodgkin lymphoma, and 3 patients with mixed-type lymphoma—at the time of diagnosis at Sun Yat-sen University Cancer Center from 2010 to 2016 (Table S1). This study was conducted in accordance with the Declaration of Helsinki. All patients and healthy controls provided written consent, and the Research Ethics Committee of Sun Yat-sen University Cancer Center (GZR2019-238) approved the study.

The NPC cell line C666 was a kind gift from Saiwah Tsao (University of Hong Kong, Hong Kong, China); the Burkitt’s lymphoma cell line AKATA (EBV positive) was maintained in our laboratory; the mature LCL cell line LCL-N15 was obtained by EBV transformation of normal resting B cells in our laboratory. Human peripheral blood mononuclear cells (PBMCs) were isolated from the blood of healthy donors. All cells were cultured in RPMI 1640 medium (Life Technologies, Beijing, China) supplemented with 10% fetal bovine serum (FBS; Gibco, Grand Island, NY, USA).

### Establishment of EBV-transformed LCLs.

Primary B cells were enriched from the peripheral blood of healthy donors by negative selection of T cells using human anti-CD3 beads (eBioscience, San Diego, CA, USA) or by positive selection using anti-CD19 beads (Stemcell Technology, Vancouver, Canada). Selected cells at 1 × 10^6^ cells/well were plated in 48-well plates (Thermo Scientific, Rockford, IL, USA), cultured with RPMI 1640 complete medium in the presence of 0.2 ng/mL cyclosporine, and treated with EBV particles. The production of EBV particles was induced from phorbol ester (30 ng/mL)-stimulated B95.8 cells according to a standard protocol, and viral production was tested by GP350 staining (46, 47). We scored EBV-transformed B-cell proliferation and colony formation at different time points either microscopically or by carboxyfluorescein diacetate succinimidyl ester (CFSE, 10 μM) labeling and flow cytometry. After at least 3 weeks of culturing, wells with clearly growing cells were defined as containing successful LCLs. To detect the influence of Gal-9, STING, STAT3, and STAT5 on the EBV-infected B-cell immortalization process, 1 μg/mL human recombinant galectin-9 protein (R&D Systems, Minneapolis, MN, USA), 1 μg/mL galectin-9 monoclonal antibody (OriGene, Rockville, MD, USA), 62.5 μg/mL STING agonist 10-carboxymethyl-9-acrianone (CMA; Sigma-Aldrich, St. Louis, MO, USA), 5 μM phosphorylated STAT3 inhibitor cryptotanshinone (Selleck, Houston, TX, USA), or 100 μM phosphorylated STAT5 inhibitor STAT5-IN-1 (MedChemExpress, New Jersey, USA) was added to the supernatant at day 0 after EBV infection and maintained during the EBV transformation process.

### RNA extraction, RNA-seq, and quantitative RT-PCR (qRT-PCR) analysis.

Total RNA from cells was extracted with TRIzol reagent (Invitrogen) according to the manufacturer’s protocol. Reverse transcription and RNA sequencing are described in the supplemental methods. The primers used in the qRT-PCR analyses are shown in Table S2. Gene-set enrichment analysis based on canonical pathways was performed as previously described (48). Briefly, the normalized expression data were analyzed and visualized using the Mfuzz and Mfuzzgui packages in R (http://mfuzz.sysbiolab.eu). The raw read counts per locus were normalized across the samples, and the differential gene expression data were analyzed using DESeq. The RNA-seq data were deposited in the NCBI Gene Expression Omnibus (GEO) under accession code GSE162516.

### Flow cytometry.

EBV-transformed B cells were stained with fluorochrome-conjugated Abs and CFSE according to the manufacturer’s instructions and then analyzed by fluorescence-activated cell sorting. In brief, cells were harvested, washed, and stained with surface phenotypic markers for 20 min on ice. Data were acquired with a Beckman Coulter Gallios flow cytometer and analyzed using FlowJo v10 software.

### Plasmid, lentivirus production, and transduction.

Gal-9-specific shRNA (shGal-9) and control shRNA (shCtrl) were cloned into the pLKO.1 vector. The plasmid encoding the full sequence of EBNA1 (vector pLNCX2) was a gift from the Musheng Zeng laboratory (SYSUCC, Guangzhou). All plasmids were confirmed by sequencing and transfected into cells using Lipofectamine 2000 (Invitrogen, Carlsbad, CA, USA).

For ribonucleoprotein (RNP) synthesis, chemically synthesized trans-activating CRISPR (tracrRNA) and CRISPR RNAs (crRNAs) were obtained from RiboBio Company, and Cas9 V3 protein preparation (1081059) was performed by Integrated DNA Technologies. The synthesis of RNP complex and nucleofection of RNP complex into B cells were performed as previously described (49). Briefly, freshly isolated primary B cells were washed and resuspended in 20 μL P3 primary cell nucleofector solution buffer according to the manufacturer’s instructions (P3 primary cell 4D-Nucleofector X kit S) and nucleofected with RNP complex using the EH100 program of the 4D Nucleofector instrument (Lonza). The transfected cells were incubated at 37°C and 5% CO_2_ for 1 h before being infected with EBV for subsequent experiments. For siRNA transduction, 10^6^ LCL in 100 μL electroporation solution were transfected with 100 μM siRNA (targeting EBNA1 or scrambled; RIBO Bio) by electroporation (Bio-Rad GenePluser Xcell, 140 V), and cells were harvested 48 h after transfection.

The sequences of crRNAs targeting LGALS9 and siRNA targeting EBNA1 were designed as follows: crRNA_Lgals9-1: ACACACATGCCTTTCCAGAA, crRNA_Lgals9-2: AACTTTCAGACTGGCTTGAC, and EBNA1 siRNA sequence: GCCAAGACATAGAGATGGT.

### Immunoblot, immunofluorescent (IF), and immunohistochemical staining and luciferase reporter assays.

The detailed description of these procedures is presented in the supplemental methods section.

### LCL tumor xenograft model in NCG mice.

Female NCG (NOD/ShiLtJGpt-Prkdc^em26Cd52^Il2rg^em26Cd22^/Gpt) mice (4 weeks old) were obtained from Yaokang Biological Technology Co., Ltd., Guangdong China. The mice were reared under specific-pathogen-free conditions and were treated in accordance with the guidelines for the use of experimental animals by the Committee on the Use of Live Animals in Teaching and Research, Sun Yat-sen University. Briefly, 5 × 10^6^ mature LCL cells suspended in 100 μL phosphate-buffered saline (PBS) were subcutaneously (s.c.) injected in the back skin of the mice. Thereafter, layered double hydroxide (LDH) nanoparticles (hexagonal and plate-like typical morphology with lateral diameters of 60 to 400 nm) (50) loaded with different plasmids were administered to the mice 3 times every 2 days. The *in vivo* treatment conditions for LDH are described below. First, the NanoLDH and shGal-9 or shCtrl plasmids were mixed at a 10:1 mass ratio, and the mixture was slowly added to a constant volume of bovine serum albumin (BSA) solution (5 times the concentration of NanoLDH). Then, the above-described solution was diluted with PBS to the specified concentration and administered to the mice by intratumor injection (group 1: LDH plus 0.5 mg/kg shGal-9 plasmid, group 2: LDH plus 0.5 mg/kg shCtrl plasmid). Tumor growth was observed until 2 weeks after the last treatment. Then, the mice were sacrificed, and after the tumor was weighed, the tumor tissues were harvested for hematoxylin and eosin (H&E) staining and further immunohistochemical (IHC) staining.

### Statistical analysis.

All statistical analyses were performed using GraphPad Prism 8 software (La Jolla, CA, USA) and SPSS 18.0 software (Chicago, IL, USA). The *in vitro* numbered results were produced from at least three independent experiments. Numerical data are presented as the means ± standard error of the mean (SEM), and statistical significance was determined using one-way analysis of variance (ANOVA) among more than two groups or a standard two-tailed Student’s *t* test or paired Student’s *t* test for two groups. For IHC scores, the cutoff values were the median of each group. Unless otherwise specified, the experiments were performed without duplicates due to material restrictions. *, *P* < 0.05; **, *P* < 0.01; *P* < 0.05 was considered significant.

### Ethics approval and consent to participate.

This study was approved by the Research Ethics Committee of Sun Yat-sen University Cancer Center (GZR2019-238). Participants gave informed consent to participate in the study before taking part. The data authenticity of this article has been validated by uploading the key raw data onto the Research Data Deposit platform (www.researchdata.org.cn) and was inspected/approved by the Sun Yat-sen University Cancer Center Data Access/Ethics Committee with approval number RDDB2022487915.

### Data availability.

The RNA-seq data were deposited in the NCBI Gene Expression Omnibus (GEO) under accession code GSE162516.
