# Supplementary material for: Galectin-9 Facilitates Epstein-Barr Virus Latent Infection and Lymphomagenesis in Human B Cells
Source: Microbiol Spectr. 2023 Jan 9;11(1):e04932-22. doi: 10.1128/spectrum.04932-22 (PMC9927364; doi:10.1128/spectrum.04932-22)
Supplement: Supplemental file 1 — Supplemental material. Download spectrum.04932-22-s0001.pdf, PDF file, 1.8 MB [file spectrum.04932-22-s0001.pdf]

Supplementary materials

Supplementary methods

RNA extraction, RNA-seq and quantitative RT–PCR (qRT–PCR) analysis

Total RNA from cells was extracted with TRIzol reagent (Invitrogen) according to the manufacturer's protocol and reverse transcribed using a Revert Aid First Strand cDNA Synthesis Kit (Invitrogen). Synthesized cDNA was quantified with ChamQ SYBR qPCR Master Mix (Vazyme, Nanjing, China). The primers used in the analyses are shown in Supplementary Table S2. RNA sequencing and analysis were conducted by RiboBio Biotechnology Company, Beijing, China. For EBV gene expression analysis, raw data were trimmed to remove adapters and low-quality sequence with Trim Galore. Then, alignments to EBV genomes (NCBI, NC_007605) was performed using bowtie2.

Immunoblot and immunofluorescent (IF) staining assays

For immunoblot assays, cells were subjected to lysis in ice-cold low-salt lysis buffer (LSB; 150 mM NaCl, 50 mM HEPES pH 7.5, 1.5 mM $MgCl_2$, 1 mM EDTA, 10% glycerol, 1% Triton X-100) supplemented with 5 mg/mL protease inhibitor cocktail (Roche, Mannheim, Germany). Aliquots of the extracts (20~25 μL) were subjected to SDS–PAGE, and the membranes were immunoblotted with the indicated antibodies. Antibodies targeting the following proteins were used: BZLF1 (Novus, Shanghai, China), LMP1 (Bioss, Beijing, China), LMP2A (Bioss), EBNA1 (Santa Cruz Biotechnology), STING (Cell Signaling Technology, Danvers, MA, USA), p-TBK1 (Cell Signaling Technology), p-STAT3 (Cell Signaling Technology), galectin-9

(Proteintech, Wuhan, China), and GAPDH (Proteintech). For IF assays, harvested EBV-transformed B cells under different treatment conditions were incubated with antibodies targeting Gal-9, STING, LMP1, BZLF1 or EBNA1 for 30 min, followed by treatment with CF488-conjugated anti-mouse antibody or CF568-conjugated anti-rabbit secondary antibody, staining with Hoechst (2 μg/ml) and fixation with 4% paraformaldehyde. Images visible under a fluorescence microscope (Leica) were acquired and observed using ImageJ software (National Institutes of Health).

Immunohistochemical staining (IHC)

IHC staining was measured using primary monoclonal anti-human Gal-9 (1:100, Proteintech), anti-human STING (1:400, Cell Signaling Technology), anti-EBNA1 antibodies (1:200, Abcam), anti-LMP1 (1:300, Santa Cruz) and anti-human CD20 (1:300, Affinity) antibodies according to the manufacturer's instructions. Gal-9 and STING expression in tumors was scored in 5 to 10 separate 400× high-power microscopic fields (HPFs). The specimens were scored based on the extent of staining as follows: 0 (0-5%), 1 (5-10%), 2 (10-50%), or 3 (>50%). EBNA1 staining data were obtained by counting the number of positively stained cells in 5 to 10 separate 400× fields. The slides were scored independently by two pathologists.

Luciferase reporter assay

The Gal-9 promoter regions (2000 bp upstream of TSA) were cloned upstream of the Gaussia luciferase gene in the pGL3-basic vector; the TK-luc plasmid (Promega,

Madison, WI, USA) was included as a control. The EBNA1-pcDNA3.1 vector was cotransfected with the Gal-9 reporter-luc or TK-luc plasmid into cells, which were lysed with lysis buffer 48 h later. The relative luciferase units (RLUs) were calculated by normalizing the Gal-9 reporter-luc reading to that of TK-luc, and the RLU value of the control cells was set to 1. The Gal-9 reporter-luc vector was constructed by JINWEIZHI Biotechnology Company (Beijing, China).

ChIP array

LCL cells were harvested and crosslinked with formaldehyde, and then a Pierce™ Magnetic ChIP Kit (Invitrogen) was used to pull down bound DNA according to the manufacturer's protocol. To aid in the lysis step, the cells were sonicated with a Qsonica Q700 for 50 cycles (each cycle included 5 seconds of active sonication followed by 20 seconds at rest) under 35 volts. For each IP reaction, we added 4 μg of anti-EBNA1 antibody (Santa Cruz Biotechnology, Dallas, TX, USA) and incubated the mixture overnight at 4°C. An anti-rabbit IgG served as a control.

Eluted DNA was analyzed by qPCR using ChamQ SYBR qPCR Master Mix (Vazyme). The following primers were used for ChIP-qPCR:

Gal-9-P1 (forward) 5′- TGCCCAGCCCCATTATTTTC -3′

Gal-9-P1 (reverse) 5′- ACTCCCAGCCCCAGTGACC -3′

Gal-9-P2 (forward) 5′- TGGGGCAATGATGAGTAAAG -3′

Gal-9-P2 (reverse) 5′- CTGGGTAATAGAGCAAGACT -3′

Chr 11.1 (forward) 5′- CCTCGTGGGTAAGCACTGTT-3′

Chr 11.1 (reverse) 5′- CCATGAGGCCACACTTATT-3′

Supplementary figures

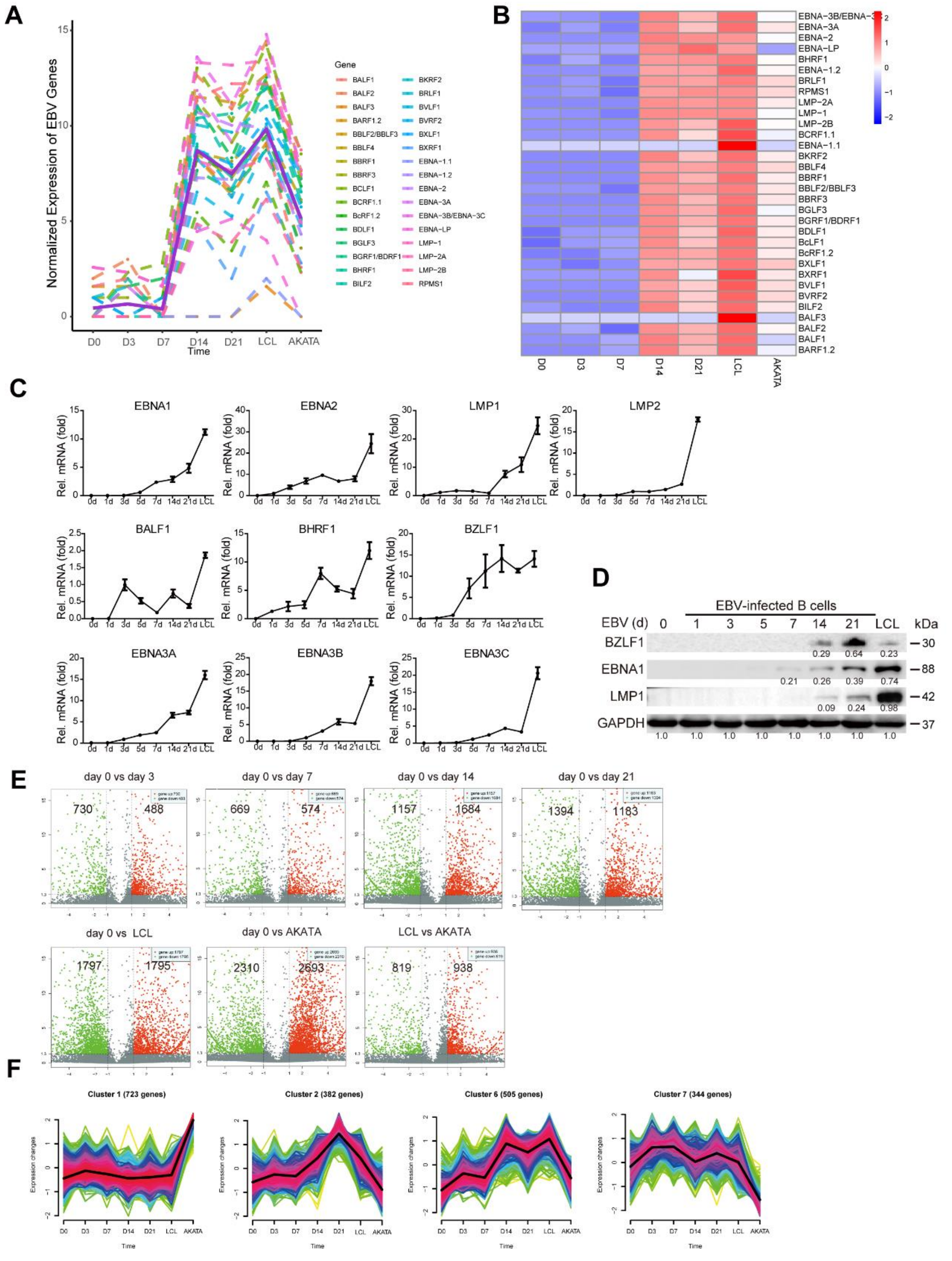

Supplementary Figure 1 related to Figure 1. Changes in the expression of EBV genes and cellular gene expression profiles during B-cell transformation (A and B). Line plot (A) and heatmap (B) of EBV genes in B cells after EBV infection and in

matured LCL and AKATA cells lines. (**C**). Quantification by quantitative real time polymerase chain reaction (qRT-PCR) analysis of viral mRNAs in EBV-infected B cells, including genes encoding lytic (BALF1, BHRF1 and BZLF1) and latent (EBNA1, EBNA2, EBNA3A, EBNA3B, EBNA3C, LMP1 and LMP2) EBV proteins, at the indicated time points after EBV infection. (**D**) Western blot detection of the EBV proteins BZLF1 (immediate early) and EBNA1, EBNA2 and LMP1 (latent) at different time points after EBV infection of B cells (data are representative of 3 independent experiments). (**E**). Volcano plots showing differentially expressed genes between native B cells (day 0) and EBV-infected B cells at the indicated time points (day 3, day 7, day 14 and day 21) or between LCL and AKATA cells by bulk RNA-seq. The differentially expressed genes were selected according to the criteria of p value < 0.05 and fold change >2. (**F**). Gene clusters were obtained from Mfuzz clustering analysis. Yellow- or green-colored lines correspond to genes with low membership value. Red- and blue-colored lines correspond to genes with high membership values.

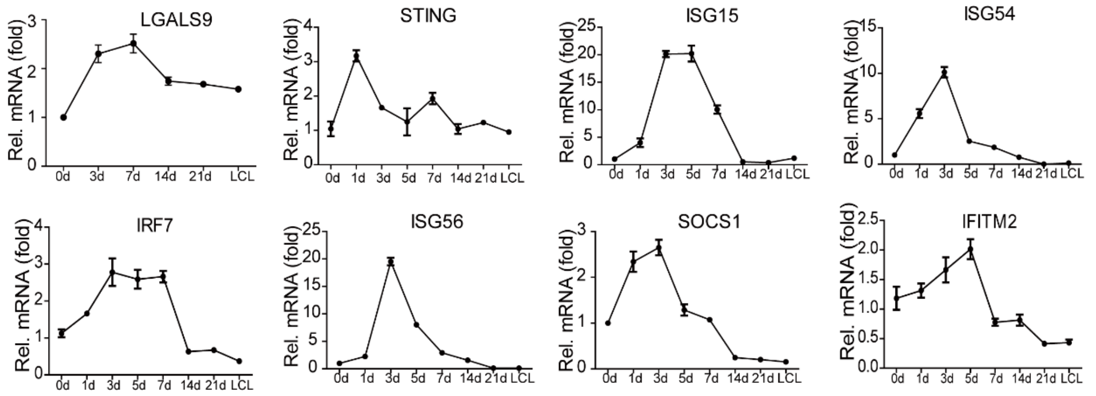

Supplementary Figure 2. Changes in the transcription levels of genes encoding biomolecules downstream of STING signaling in the process of EBV-infected B-cell transformation. Quantitative assessment by real-time PCR (qRT–PCR) of the mRNA levels of genes encoding biomolecules downstream of LGALS9 and STING signaling, including STING, ISG15, ISG54, IRF7, ISG56, SOCS1 and IFITM2.

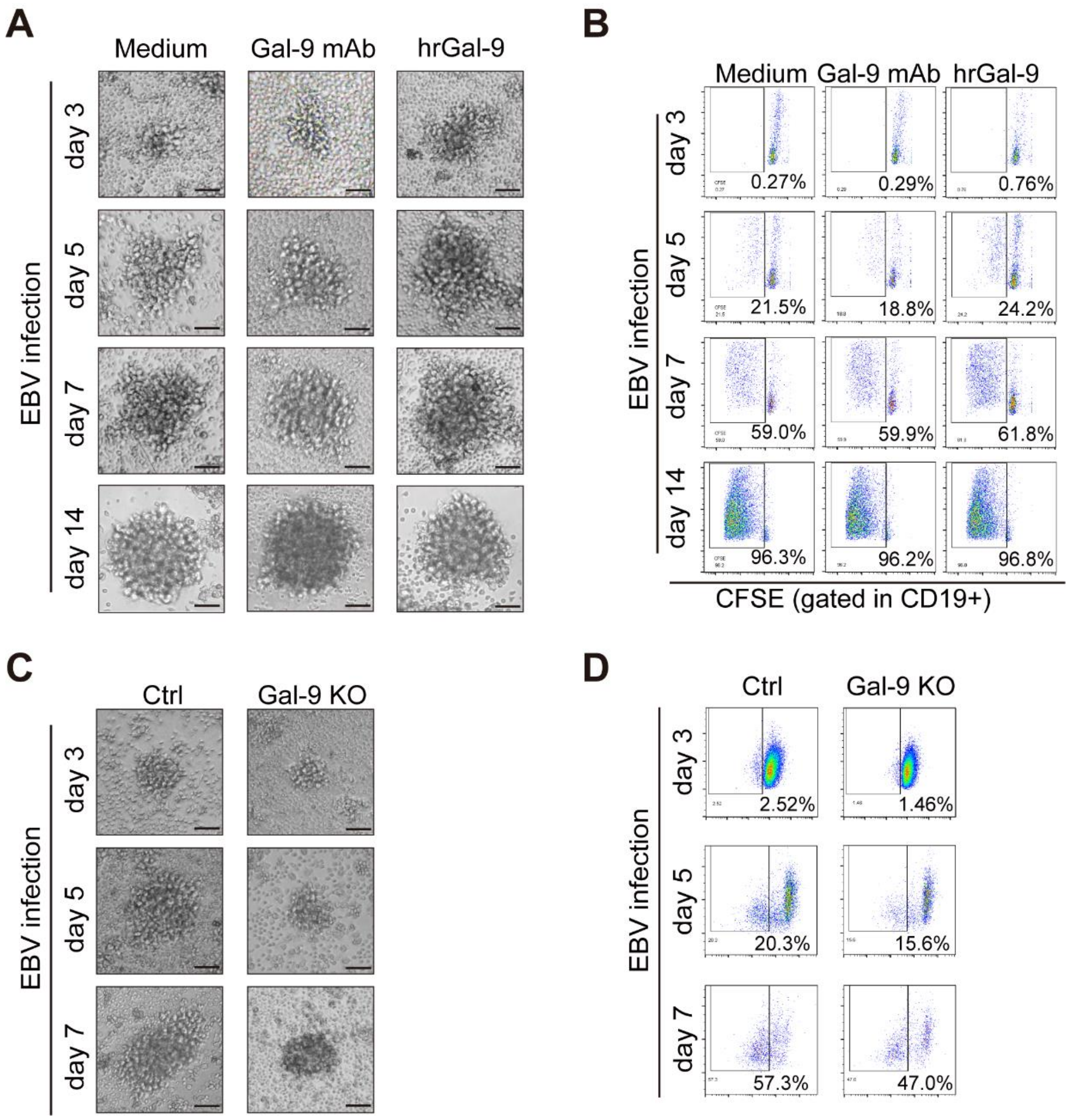

Supplementary Figure 3. Gal-9 promotes cell proliferation at the early stage of EBV infection. (**A**) Representative images of colonies formed and (**B**) flow cytometry assay showing the proliferation of CFSE-labeled CD19+ cells at the indicated time points after EBV infection in the presence of vehicle only (medium), hrGal-9 protein alone or anti-Gal-9 neutralizing antibody alone. (**C**) Representative images of colonies formed and (**D**) flow cytometry assay showing the proliferation of CFSE-labeled CD19+ cells at the indicated time points after EBV infection in Gal-9 KO and Ctrl EBV-infected B cells. Scale bar: 20 μm.

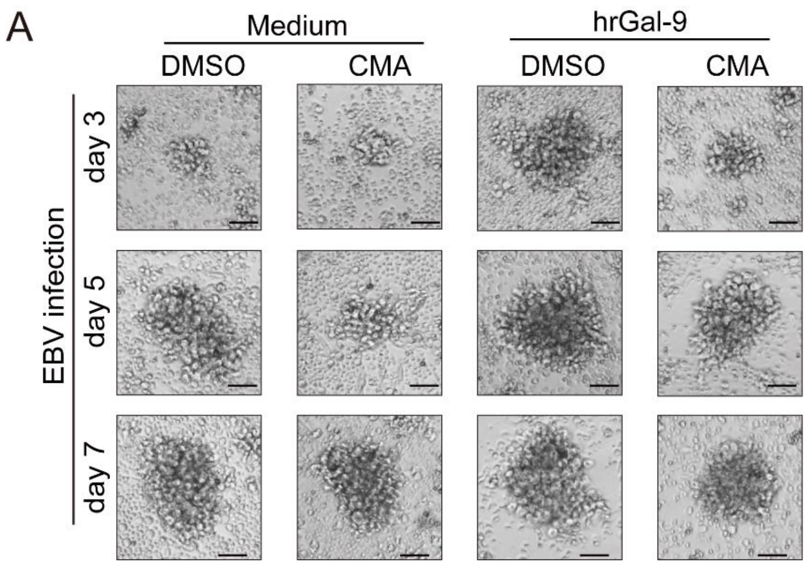

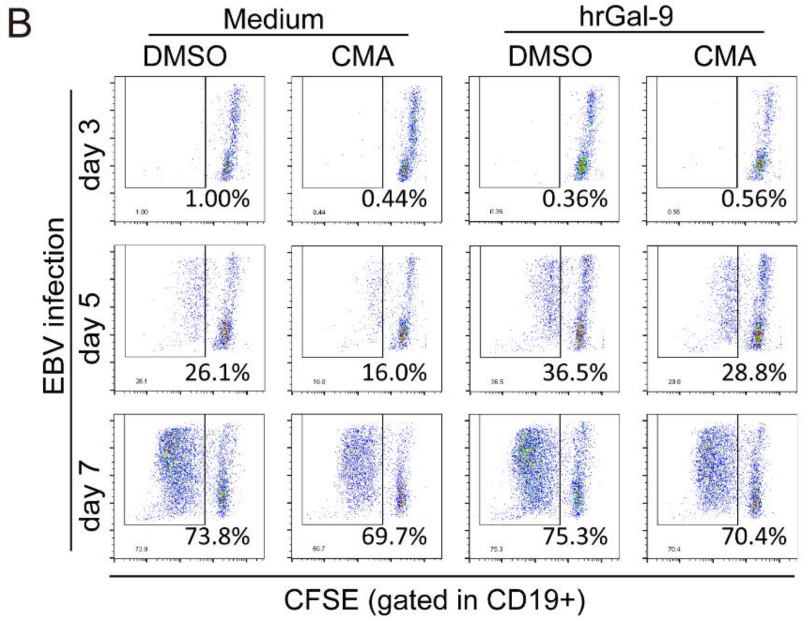

Supplementary Figure 4. Activation of STING signaling suppresses the proliferation of EBV-infected B cells (A) Representative images of EBV-infected B cells under the indicated treatments: DMSO alone, CMA alone, DMSO + hrGal-9, or CMA + hrGal-9. Images under a phase contrast microscope (100X magnification) were recorded at the indicated time points. Scale bar: 20 μm. (B) Flow cytometry assay showing the proliferation of CSFE-labeled CD19+ cells at the indicated time points after EBV infection in response to the indicated treatments.

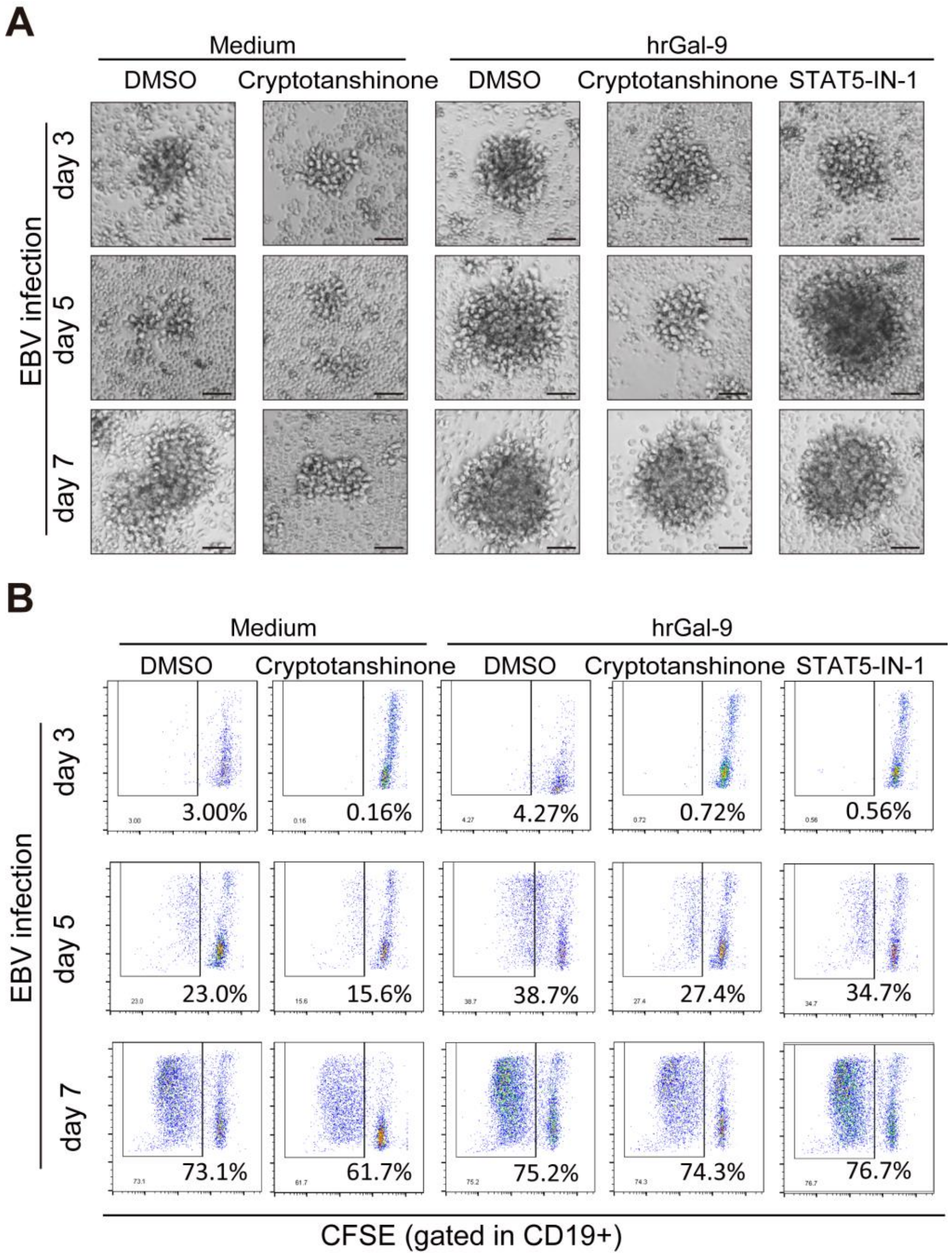

Supplementary Figure 5. Inhibition of STAT3 signaling reduces the proliferation of infected B cells independent of Gal-9 treatment. (A) Representative images of EBV-infected B cells under the indicated treatments: cryptotanshinone (a STAT3 inhibitor) alone, hrGal-9 alone, cryptotanshinone + hrGal-9, or hrGal-9 + STAT5-IN-1 (a STAT5 inhibitor). Images under a phase contrast microscope (100X magnification) were recorded at the indicated time points. Scale bar: 20 μm. (**B**) Flow cytometry assay

showing the proliferation of CSFE-labeled CD19+ cells of EBV-infected B-cell colonies in each of the above-listed experimental conditions.

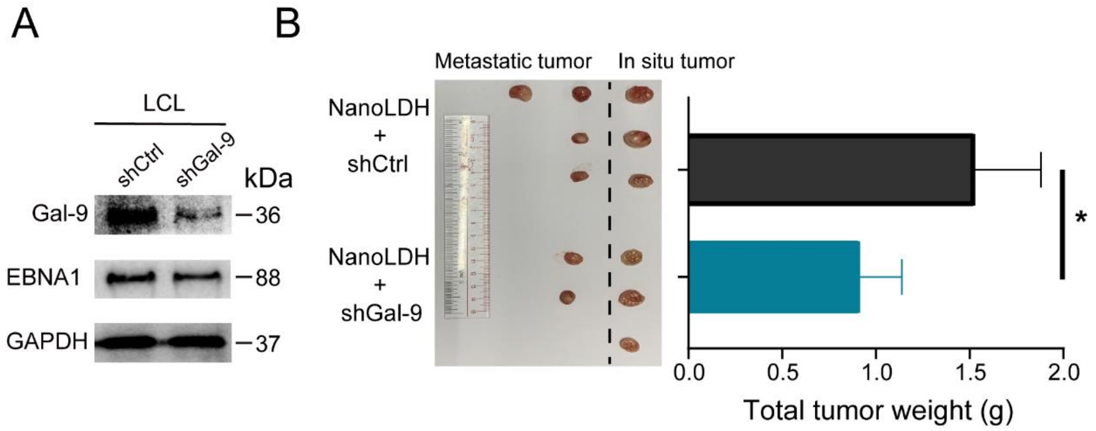

Supplementary Figure 6. Treatment with NanoLDH loaded with shGal-9 reduced the weight of xenografted LCL tumors in NCG mice (A). The protein expression of Gal-9 and EBNA1 in LCL with NanoLDH + shCtrl or NanoLDH + shGal-9 treatment in vitro. (**B**). Images and tumor weight of in situ and metastatic tumors from mice receiving intratumor NanoLDH + shCtrl or NanoLDH + shGal-9 treatment when the mice were sacrificed. The statistical significance of (B) was determined by an unpaired Student's t test; *p < 0.05, **p < 0.01 and p < 0.05 were considered significant.

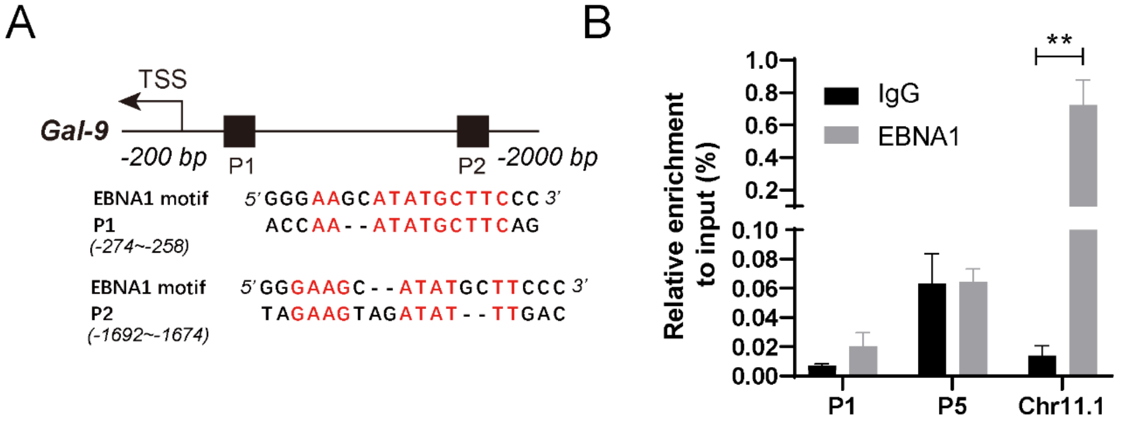

Supplementary Figure 7. The transcript regulation of Gal-9 by EBNA1. (**A**). DNA segments identified as potential EBNA1 target sequences upstream of the transcription start site (TSS) of the Gal-9 gene (-200 to -2000 nucleotides) and sequences of putative EBNA1 binding sites (the nucleotides in red color). (B). Validation of EBNA1 binding to the Gal-9 promoter sequence by ChIP-qPCR. Comparisons between two groups were conducted with an unpaired Student's t test. *$p < 0.05$, **$p < 0.01$ and $p < 0.05$ were considered significant. Chr11.1 represents the motif found in the chromosome 11 cluster, here as the positive control of the EBNA1 binding site.

Supplementary Tables

Supplementary Table 1. Clinical characteristics of BCL patients

**Characteristic**	**No. (%) of patients**
**Total cases**	66 (100%)
**Age**	
Range	3-89
Median	56
Average	48.42
**Gender**	
Male	34 (51.5%)
Female	32 (48.5%)
**EBER**	
+	31 (47.0%)
-	35 (53.0%)
**Subtype at diagnosis**	
**Non-Hodgkin lymphoma**	54 (81.8%)
Diffuse large B cell	28 (42.4%)
Burkitt's	19 (28.8%)
Other types[a]	7 (10.6%)
**Hodgkin lymphoma**	9 (13.6%)
**Miscellaneous[b]**	3 (4.5%)
**Clinical parameters**	
**Ann Arbor stage**	Total 43
I	6 (14.0%)
II	11 (25.6%)
III	7 (16.3%)
IV	19 (44.2%)
**Death**	Total 36
No	24 (66.7%)
Yes	12 (33.3%)
**DFS**	Total 36
≤ 39 months	18 (50.0%)
> 39 months	18 (50.0%)

Note: Thirty of the 66 patients were outpatients, and some of the following data, including Ann Arbor stage information, were missing for the 30 outpatients.

a: Other types of non-Hodgkin lymphomas, excluding diffuse large B-cell lymphoma and Burkitt's lymphoma;

b: various Hodgkin and non-Hodgkin lymphomas.

Supplementary Table 2. Primer sequences

Galectin-9-F	5′-GGACGGACTTCAGATCACTGT-3′
Galectin-9-R	5′-CCATCTTCAAACCGAGGGTTG-3′
EBNA2-F	5′-AGGCTGCCCACCCTGAGGAT-3′
EBNA2-R	5′-GCCACCTGGCAGCCCTAAAG-3′
BZLF1-F	5′-GGGGCTAACCAAGGACAACA-3′
BZLF1-R	5′-ATTCCTCCAGCGATTCTGGC-3′
BRLF1-F	5′-AGAGGATCAGGCCCTTCCAT-3′
BRLF1-R	5′-AACAGATGACTTGCCTCGGG-3′
BALF1-F	5′-ATAGGAAGCTGTACGCGGAG-3′
BALF1-R	5′-ACCGCAAACACCACTGTGTA-3′
BPLF1-F	5′-GGGGAAAAGGGGAGCACTAC-3′
BPLF1-R	5′-AGGAGGTAGGAGACAGCCAG-3′
BHRF1-F	5′-TATACGGGACAGTCGTGTGC-3′
BHRF1-R	5′-AACTACAGTGTCCTCTGGCG-3′
CD21-F	5′-TCTGTGTGTTGAAGGGCAGG-3′
CD21-R	5′-TGTGGATCATAGGAAGTGCTGG-3′
NRP1-F	5′-ACGTGGAAGTCTTCGATGGAG-3′
NRP1-R	5′-CACCATGTGTTTCGTAGTCAGA-3′
NMHC-IIA-F	5′-CAGCAAGCTGCCGATAAGTAT-3′
NMHC-IIA-R	5′-CTTGTCGGAAGGCACCCAT-3′
EBF1-F	5′-TGGGGTTCGTGGAGAAGGAA-3′
EBF1-R	5′-CACGTAGAAATCCTGCTCCG-3′
IL6R-F	5′-CCCCTCAGCAATGTTGTTTGT-3′
IL6R-R	5′-CTCCGGGACTGCTAACTGG-3′
MEF2B-F	5′-CGGGCCCCTGATCTTCG-3′
MEF2B-R	5′-CGTCACCTGCCGATTCCTTT-3′
EBNA1-F	5′-CAACCTCAGGCGAGGAATTT-3′
EBNA1-R	5′-CCTTAGTGGGCCAGGTTGT-3′
LMP1-F	5′-CCCTTTGTATACTCCTACTGATGATCAC-3′
LMP1-R	5′-ACCCGAAGATGAACAGCACAAT-3′
LMP2-F	5′-TTCCTCATTGGCTTTGCCCT-3′
LMP2-R	5′-AGTGTTGCGATATGGGGTCG-3′
IFIT1-F	5′-AGAAGCAGGCAATCACAGAAAA-3′
IFIT1-R	5′-CTGAAACCGACCATAGTGGAAAT-3′
IFITM2-F	5′-GCTCACTGAGAACCATCCCG-3′
IFITM2-R	5′-TGTGGATCACGGTGGACATC-3′
IRF7-F	5′-CCCACGCTATACCATCTACCT-3′
IRF7-R	5′-GATGTCGTCATAGAGGCTGTTG-3′
EBNA3A-F	5′-AGCGACCAGTTTACCCCAAG-3′
EBNA3A-R	5′-CATCTGACTGGGAGAACGGG-3′
EBNA3B-F	5′-AACTTAGCAGTTCGGCACCA-3′

EBNA3B-R	5′-CGCATAGGGATAGGTCGCAG-3′
EBNA3C-F	5′-AAAGATACGCTCGGGAAGCC-3′
EBNA3C-R	5′-CCTCGGTCTGGAGCAAGTTT-3′
STING-F	5'-CCAGAGCACACTCTCCGGTA-3'
STING-R	5'-CGCATTTGGGAGGGAGTAGTA-3'
ISG54-F	5'-GGAGGGAGAAAACTCCTTGGA-3'
ISG54-R	5'-GGCCAGTAGGTTGCACATTGT-3'
SOCS1-F	5'-TTTTCGCCCTTAGCGTGAAGA-3'
SOCS1-R	5'-GAGGCAGTCGAAGCTCTCG-3'
ISG15-F	5'-TCCTGGTGAGGAATAACAAGGG-3'
ISG15-R	5'-GTCAGCCAGAACAGGTCGTC-3'
